# Evaluation of Long-Read RNA Sequencing Procedures for Novel Isoform Identification and Quantification in Human Whole Blood

**DOI:** 10.3390/genes16091075

**Published:** 2025-09-12

**Authors:** Hikari Okada, Alessandro Nasti, Yoshio Sakai, Yumie Takeshita, Sadahiro Iwabuchi, Ho Yagi, Tomomi Hashiba, Noboru Takata, Taka-Aki Sato, Takeshi Urabe, Seiji Nakamura, Toshinari Takamura, Taro Yamashita, Takuro Tamura, Kenichi Matsubara, Shuichi Kaneko

**Affiliations:** 1 Information-Based Medicine Development, Graduate School of Medical Sciences, Kanazawa University, 13-1 Takara-machi, Kanazawa 920-8641, Japan; 2 Department of Gastroenterology, Kanazawa University Hospital, 13-1 Takara-machi, Kanazawa 920-8641, Japan; 3 Department of Endocrinology and Metabolism, Graduate School of Medical Sciences, Kanazawa University, 13-1 Takara-machi, Kanazawa 920-8640, Japan; 4 Department of Bioinformatics and Genomics, Graduate School of Medical Sciences, Kanazawa University, 13-1 Takara-machi, Kanazawa 920-8640, Japan; 5 iLAC Co., Ltd., Tsukuba 305-0821, Japan; 6 Department of Gastroenterology, Public Central Hospital of Matto Ishikawa, 3-8 Kuramitsu, Hakusan 924-8588, Japan; 7 DNA Chip Research Inc., Tokyo 105-0022, Japan; 8 Research and Development Center for Precision Medicine, University of Tsukuba, Tsukuba 305-8550, Japan

**Keywords:** human whole blood, long-read RNA sequencing, T2T-CHM13, GRCh38, isoform identification, isoform expression

## Abstract

Background/Objectives: Blood flows through the body and reaches all tissues, contributing to homeostasis and physiological functions. Providing information and understanding on how the transcriptome of whole blood behaves in response to physiological or pathological stimuli is critical. Methods: We collected blood from four healthy individuals and performed long-read RNA sequencing (lrRNA-seq) for the precise identification and expression quantification of RNA variants. Moreover, we compared two genome references: the Genome Reference Consortium Human Build 38 (GRCh38) and the Telomere-to-Telomere (T2T) assembly of the CHM13 cell line (T2T-CHM13). Results: With GRCh38, we could identify an average of about 46,000 genes, 1.3-fold more genes than T2T-CHM13. Similarly, we identified about 185,000 isoforms with GRCh38 and 140,000 with T2T-CHM13, finding similar differences for full splice match (FSM) and incomplete splice match (ISM) transcript isoforms. There were about 90,000 novel isoforms for GRCh38 and 70,000 for T2T-CHM13, 47% and 50% of the total number of identified isoforms, respectively. Differences in isoform numbers between GRCh38 and T2T-CHM13 were identified for the subcategories “Genic Genomic”, “Intergenic”, and “Genic Intron”. Using GRCh38, we generally identified a higher number of non-coding isoforms, as well as a higher number of isoforms aligning within intron and intergenic regions. Nonetheless, GRCh38 might incur false positive results, and T2T-CHM13 is likely more accurate for genome sequences in the repetitive regions. Conclusions: LrRNA-seq is a valid method for the identification of novel isoforms in blood, and this study is a first step toward the creation of a comprehensive database of the structure and expression of transcript isoforms for optimized predictive medicine.

## 1. Introduction

Blood flows through the body and reaches all peripheral tissues. It contributes to body homeostasis and maintains physiological functions. We reported previously that, in the long-term gene expression profiles of whole blood of patients undergoing regular medical health check-ups, a group of genes was stably expressed over time. These genes were mainly involved in immune system pathways, including antigen cell presentation and interferon-related signaling; meanwhile, in another group of genes, we observed altered gene expression related to altered cellular machinery signaling [1].

This longitudinal examination showed that the whole blood gene expression signature can reveal unmanifested physiological changes. Moreover, it is now possible to predict the presence or absence of disease from RNA information in the blood. However, the accuracy of disease prediction is questionable because the basis of RNA information in the blood is incomplete. We focused on the long-read RNA-seq method, which provides more accurate isoform RNA information than short-read RNA-seq [2]. Moreover, since many isoforms are still undiscovered, lrRNA-seq is a powerful method to identify new isoforms [3,4]. The large number of variants contribute to the intricacy of the transcriptome, influenced by mechanisms such as RNA editing, alternative splicing, and alternative polyadenylation. RNA isoforms with unique structural properties, functionality, and stability are generated [5,6].

The identification, characterization, and quantification of these variants can reveal how full blood gene expression behaves in physiological settings and under different pathological conditions [3]. Based on this background, we collected full blood from four individuals who underwent medical health checkups, and analyzed their overall RNA variant composition and expression using PacBio lrRNA-seq. Because lrRNA-seq can technically read RNA sequences larger than 1 kb, it can detect RNA information with high accuracy. However, the ability of RNA-seq to efficiently identify and quantify gene expression depends on the comprehensiveness of the selected genomic reference. In this regard, we focused on the comparison between two genome references, the Genome Reference Consortium Human Build 38 (GRCh38) [7] and the Telomere-to-Telomere (T2T) assembly of the CHM13 cell line (T2T-CHM13) [8,9]. GRCh38 has been widely used [10], is well annotated, and is used as the standard tool for many existing data [11,12]. The T2T-CHM13 reference provides more accurate genome sequences, including repetitive regions, such as telomeres and centromeres [13], newly added and corrected 238 Mb of sequence, and 1956 new genes [8,14]. In principle, GRCh38 should be chosen for comparison with existing datasets, while T2T-CHM13 should be chosen when focusing on the analysis of new genes and repetitive regions [14,15,16].

Where possible, it would be best to analyze using both references and compare the results. At present, the appropriateness of the reference may vary depending on the research purpose and analysis target. T2T-CHM13 is likely to become more widespread in the future, and a transition to T2T-CHM13 is expected in the long term [14,17]. Regardless of the reference chosen, it is important to clearly record the version of the used reference genome and consider it when interpreting results and comparing them with other studies. Few studies used whole blood for whole transcriptome analysis using long-read RNA-seq, and only two papers used GRCh38 as a reference [18,19]. Whole blood was also used as the source of RNA for another two studies, but in these cases, the target genes of RNA in whole blood had already been determined, and only a limited portion of the RNA was analyzed [20,21]. In addition, previous long-read RNA-seq annotations have used either the GRCh38 reference, or, in a limited number of studies, a reference that integrates both GRCh38 and T2T-CHM13 genome assemblies [14,22,23]; however, these studies used preestablished cell lines or deposited datasets for analysis, and none examined whole blood samples.

Against this background, this is the first comparative study using long-read RNA-seq from whole peripheral blood and data processed using GRCh38 and T2T-CHM13 genomic references for the whole transcriptome. Our future objective is to identify the RNA isoforms present in healthy human adult blood samples to create a database that can show transcript isoform variations when whole human blood is used as a source. This preliminary study is a necessary step toward building such a platform for optimized predictive medicine.

## 2. Materials and Methods

### 2.1. Study Design

In this study, we withdrew by standard phlebotomy the peripheral whole blood from 4 participants (males: *n* = 2; females: *n* = 2. 1_M36, 2_M_105, 3_M127, 4_M166), who visited the Public Central Hospital of Matto Ishikawa for medical examinations in 2016, as previously described [1,24]. The participants, who were considered healthy and had no apparent disease, were informed about the study, and their consent was obtained. Clinical parameters were recorded for all 4 individuals (Supplementary Table S1). The study protocol information and ethical approvals are described in the “Institutional Review Board Statement” section.

### 2.2. Whole Blood RNA Isolation and lrRNA-Seq Library Creation

Patient whole blood was collected in 2016 and stored in PAXgene Blood RNA Tubes, IVD (762165, QIAGEN, Hilden, Germany). Total RNA was extracted from stored blood according to the PAXgene Blood RNA Kit (762164, QIAGEN) protocol. A total RNA quality check was performed according to the Agilent RNA 6000 Nano Kit (5067-1511, Agilent Technologies, Inc., Santa Clara, CA, USA) protocol and analyzed using an Agilent 2100 Bioanalyzer (Agilent Technologies, Inc.). After confirming that the RIN value of RNA was ≥7, cDNA was synthesized and amplified using the Iso-Seq Express 2.0 kit (103-071-500, PacBio, Menlo Park, CA, USA). The prepared cDNA was then ligated with an SMRT adapter at the 5′ and 3′ ends using the SMRTbell prep kit 3.0 (102-182-700, PacBio). After library preparation, the cDNA libraries were sequenced on a Sequel IIe system (PacBio). The numbers of total reads for samples 1_M36, 2_M_105, 3_M127, and 4_M166 were, respectively: 4,456,301; 4,544,423; 4,288,374; and 4,245,914.

### 2.3. Sequence Analysis Processing

Raw PacBio Iso-seq data (BAM files) were aligned to the GRCh38 or T2T-CHM13 genome (GCA_009914755.4 [T2T-CHM13v2.0]) using pbmm2 v10.0 (Release Version 1.10.0 · PacificBiosciences/pbmm2 · GitHub). Isoform analysis was performed with Isoseq v4.0.0 (https://github.com/PacificBiosciences/IsoSeq, accessed on 13 October 2023) and classification of full-length isoforms with Pigeon v1.0.0 (https://isoseq.how/classification/pigeon.html, accessed on 12 December 2022) from the aligned data was performed. Sequence analysis processing was performed at the Research and Development Center for Precision Medicine, Tsukuba University. SQANTI3 (ver. 5.0, GitHub—ConesaLab/SQANTI3: Tool for the Quality Control of Long-Read Defined Transcriptomes) was used by the analysis pipeline of Bioengineering Lab. Co. (Sagamihara, Japan). The isoforms were annotated with gene transfer format (GTF) files downloaded from the UCSC Genome Browser [10]: for HG38, https://hgdownload.soe.ucsc.edu/goldenPath/hg38/bigZips/genes/hg38.ncbiRefSeq.gtf.gz (accessed on 29 February 2024); for T2T, https://hgdownload.soe.ucsc.edu/goldenPath/hs1/bigZips/genes/hs1.ncbiRefSeq.gtf.gz (accessed on 29 February 2024). Both GRCh38 and T2T-CHM13 genome references can be downloaded from the NCBI. Users should note that genomic coordinates and annotations differ between the two references, and mapping and annotation should be performed consistently to ensure reproducibility [14]. The mRNA transcript isoforms were filtered for a count value of at least 2 unique molecules per million of reads. All plots were created by using Origin, Version 2021 (OriginLab Corporation, Northampton, MA, USA), except if stated otherwise.

## 3. Results

### 3.1. Known and Novel Isoforms Identified in Whole Blood

**Figure 1:**
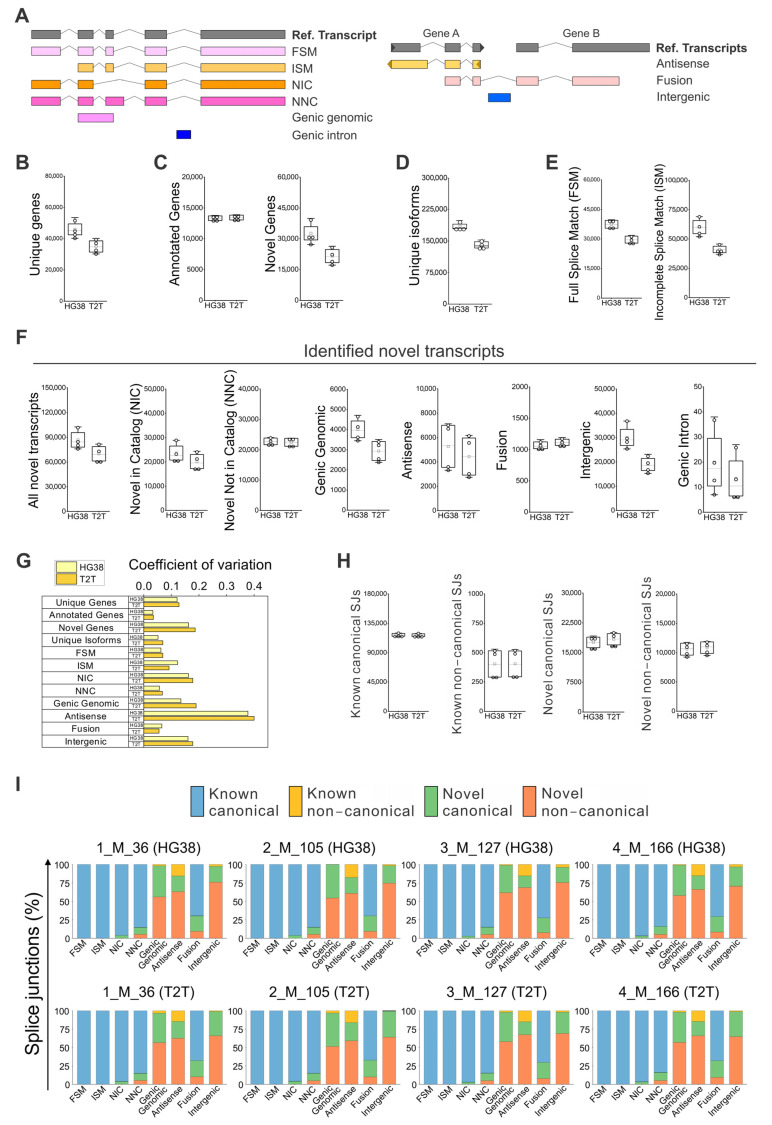
Identification of known and novel genes and isoforms in peripheral blood using GRCh38 (HG38) and T2T-CHM13 (T2T) genome references. (**A**) SQANTI classification for isoforms [25,26], briefly: Full Splice Match (FSM), Incomplete Splice Match (ISM), Novel in Catalog (NIC), Novel Not in Catalog (NNC), Genic genomic, Genic intron, Antisense, Fusion, and Intergenic. Iso-Seq data analyzed with GRCh38 and T2T-CHM13 references for the identification of: (**B**) all unique genes, (**C**) annotated and novel genes within all unique genes, (**D**) identified unique isoforms; and (**E**) FSM and ISM isoforms. (**F**) Identified novel isoforms, NIC, NNC, antisense, fusion, genic genomic, intergenic, and genic intron isoforms. (**G**) Coefficient of variation (CV) calculated for each type of isoform (**A**–**F**) for determining the distribution variability of detected isoforms among individuals, which is a value indicative of physiological diversity among individuals. The coefficient of variation was defined as the ratio of the standard deviation to the average of distribution [27]. (**H**) Absolute numbers of “Known canonical SJs”, “Known non-canonical SJs”, “Novel canonical SJs”, and “Novel non-canonical SJs” quantification by GRCh38 and T2T-CHM13. (**I**) Detail of splice junctions’ percentage by type of SJs for known and novel subcategories of isoforms. (**B**–**F**,**H**) N = 4; white boxes show the interquartile range (25–75% percentile range), the median is designated by the horizontal line, and the mean is indicated by the white square. Boundaries of the whiskers are based on the 1.5 interquartile range value, and all observed data points outside the limit of the whiskers are considered outliers.

The clinical parameters for all four individuals (Supplementary Table S1) did not show major health concerns, and they were considered disease-free. Full blood was used for the identification and quantification of known and novel isoforms by long-read RNA sequencing. The differences and similarities among the four samples were depicted by PCA analysis using either GRCh38 or T2T-CHM13 (Supplementary Figure S1). The samples were approximately equidistant and evenly distributed across the PCA plot, with no distinct clusters, indicating that each sample exhibited unique transcriptomic variability. The SQANTI classification categorizes the transcript isoforms based on splice junctions (SJs) [25,26], briefly (Figure 1A): isoforms matching precisely the reference isoforms are identified as full splice match (FSM), while isoforms labeled as incomplete splice match (ISM) have matching consecutive splice junctions, as well as missing splice junctions at the 5′, 3′, or both ends [26]. New isoforms of already known genes are categorized as Novel in Catalog (NIC), containing known splice junctions or new splice junctions from known donors and acceptors: Novel Not in Catalog (NNC) with new donors and/or acceptors [25]. Genic genomic isoforms partially overlap exons and an intron in an annotated gene, genic intron isoforms are located within an intron, Antisense isoforms overlap with coding sequences on the opposite strand of the annotated gene, Fusion isoforms bridge two annotated loci, and Intergenic isoforms do not overlap with the genomic interval spanned by an annotated gene (Figure 1A).

We found that the Iso-Seq whole blood data analyzed with GRCh38 could identify about 46,000 genes, 1.3-fold more genes than T2T-CHM13 (Figure 1B). This difference is not due to the identified known genes, but to the difference in identified novel genes (Figure 1C). Moreover, a similar increase was confirmed for unique isoforms (Figure 1D)—about 185,000 isoforms were identified with GRCh38 versus 140,000 isoforms with T2T-CHM13. This difference was observed for FSM and ISM (Figure 1E) isoforms, respectively, with a ratio of about 1.3- and 1.5-fold in favor of GRCH38. The total number of novel isoforms was about 90,000 for GRCh38 and 70,000 for T2T-CHM13 (Figure 1F). Of these, 47% were the novel isoforms identified with GRCh38, and 50% were the novel isoforms identified with T2T-CHM13. When we analyzed the average numbers of novel isoforms, similar amounts were identified for “NIC”, “NNC”, “Antisense”, and “Fusion”, while the largest difference in isoform numbers between GRCh38 and T2T-CHM13 were identified for the subcategories “Genic Genomic”, “Intergenic”, and “Genic Intron”. When we focused on the variability of the identified numbers of isoforms among individuals, a value indicative of physiological differences, we observed a high degree of variation in the number of “Antisense” isoforms, independent of the reference genome used (Figure 1G), followed also by a moderate level of variation in “NIC”, “Genic Genomic”, and “Intergenic” isoforms.

### 3.2. Comparison of Splice Junction Profiles and Isoform Characteristics Using GRCh38 and T2T-CHM13 References in SQANTI Analysis

SQANTI identifies isoforms based on their splice junctions—the base pair GT at the beginning and AG at the end of the intron is located in 98.9% of all introns in the human genome [28]. Specifically, GT–AG, GC–AG, and AT–AC are canonical splicing acceptor/donor pairs found in most human introns [28,29], while the remaining combinations are defined as non-canonical splice junctions [25]. In our data, no difference was observed between GRCh38 and T2T-CHM13 for the absolute numbers of “Known canonical SJs”, “Known non-canonical SJs”, “Novel canonical SJs”, and “Novel non-canonical SJs” (Figure 1H). When we verified in detail the splice junctions percentage by type of SJs, GRCh38 and T2T-CHM13 performed similarly (Figure 1I); among patients, FSM, ISM, and NIC isoforms were characterized mainly by “Known canonical SJs”; “NNC”, and “Fusion” isoforms by about 70–80% of “Known canonical SJs”; and the remainder by “Novel canonical SJs” and “Novel non-canonical SJs”. “Genic Genomic”, “Intergenic”, and “Antisense” isoforms all had a high percentage of “Novel canonical SJs” and “Novel non-canonical SJs”. In the case of “Antisense” variants, we also detected about 20% of “Known non-canonical SJs” (Figure 1I).

**Figure 2:**
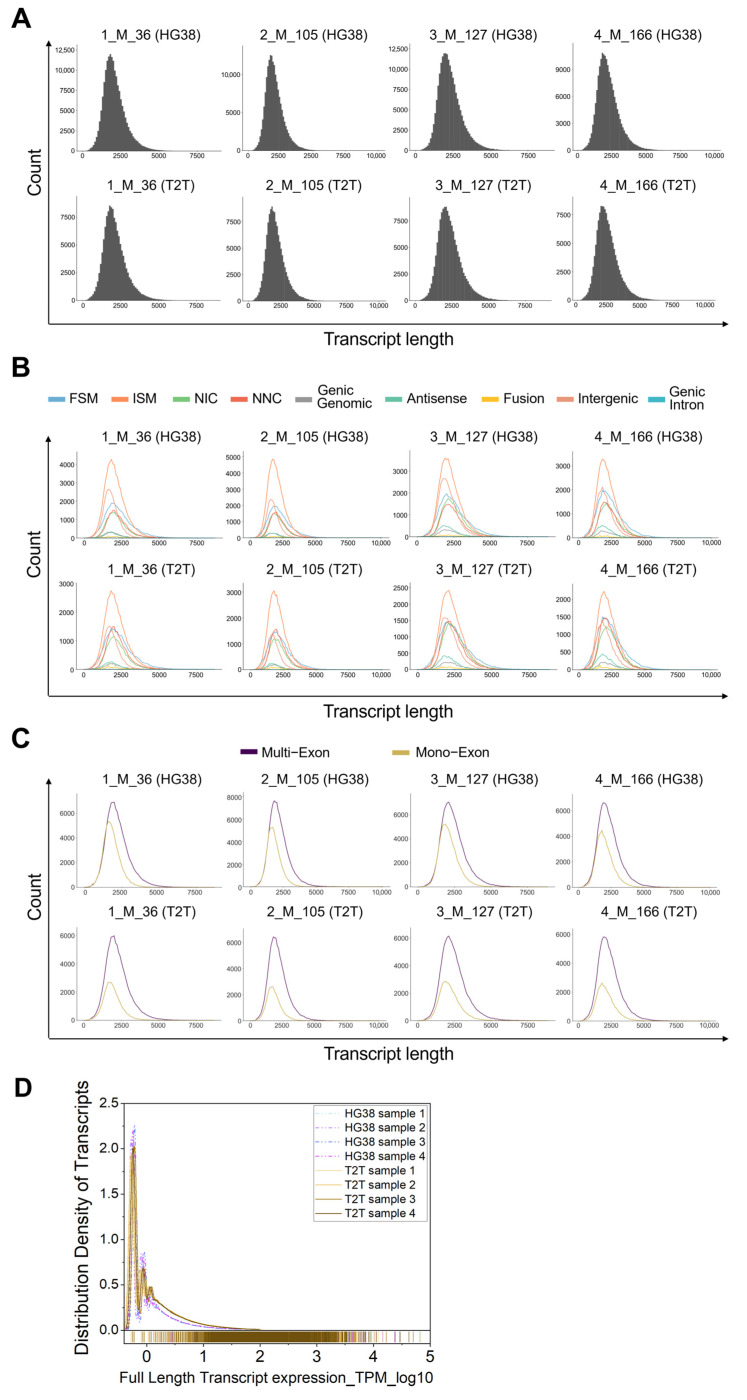
Count quantification per isoform length in peripheral blood using GRCh38 (HG38) and T2T-CHM13 (T2T) genome references. (**A**) Reading counts for all isoform length distributions for each sample, HG38 versus T2T-CHM13. (**B**) Reading counts of isoforms for each subcategory among individuals by GRCh38 and T2T-CHM13 references. (**C**) Analysis of reading counts of “Multi-Exon” and “Mono-Exon”. (**A**–**C**) The plots were depicted as obtained from the SQANTI3 output. (**D**) Distribution density of isoforms when GRCh38 or T2T-CHM13 were used for each sample.

No difference was observed between individuals regarding the read counts for all isoform length distributions (Figure 2A). However, in general, the GRCh38 number of counts per isoform was higher than the one obtained with T2T-CHM13. Moreover, we confirmed a difference in the read counts of the “ISM” and “Intergenic” variant subcategories among individuals based on the GRCh38 reference, whereas we did not see the same difference when we used T2T-CHM13 (Figure 2B). Finally, in the analysis of read counts of the categories “Multi-Exon” and “Mono-Exon”, we confirmed a higher number of counts for “Mono-Exon” variants when the GRCh38 reference was used (Figure 2C). A similar overall pattern was confirmed for the distribution density of isoforms when GRCh38 or T2T-CHM13 were used (Figure 2D).

### 3.3. Features of Coding and Non-Coding Isoforms

**Figure 3:**
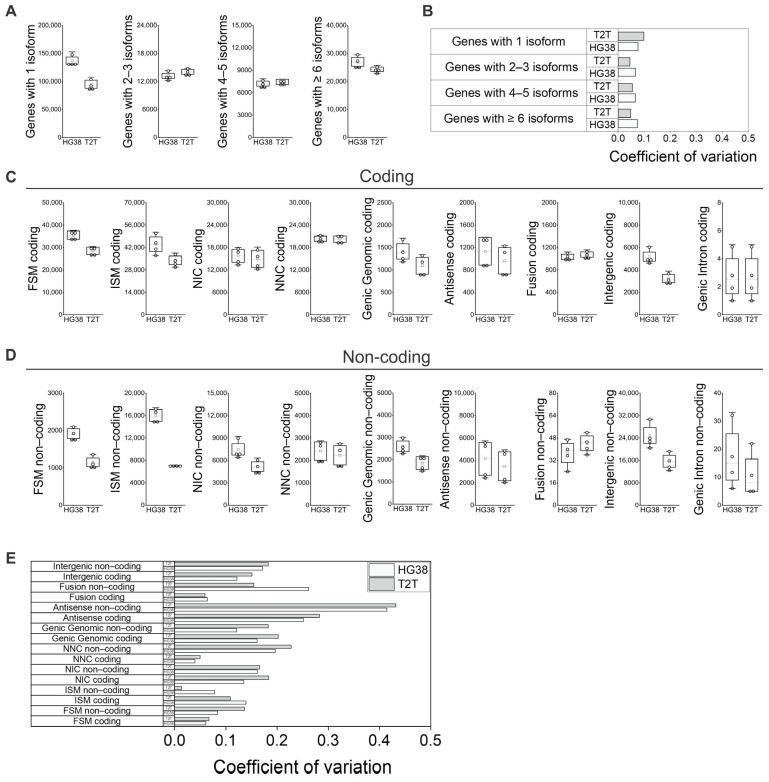
Features of coding and non-coding isoforms in peripheral blood using GRCh38 (HG38) and T2T-CHM13 (T2T) genome references. (**A**) Number of isoforms per gene; (**B**) variability of number of isoforms per gene among samples for both GRCh38 and T2T-CHM13 references. (**C**) Number of coding isoforms by subcategories, and (**D**) number of non-coding isoforms by subcategories. (**E**) Identified isoform number variability among individuals in the context of differences between “Coding” and “Non-coding” subcategories for both GRCh38 and T2T-CHM13 references. (**A**,**C**,**D**) N = 4; white boxes show the interquartile range (25–75% percentile range), the median is designated by the horizontal line, and the mean is indicated by the white square. Boundaries of the whiskers are based on the 1.5 interquartile range value; all observed data points outside the limit of the whiskers are considered outliers. (**B**,**E**) The coefficient of variation was defined as the ratio of standard deviation to the average of distribution [27].

When we analyzed the number of isoforms identified per gene, we found the main difference between GRCh38 and T2T-CHM13 to be in the number of genes with 1 isoform, while a similar number of isoforms were obtained for those genes producing 2–3, 4–5, or more than 5 isoforms (Figure 3A). Variability of the number of isoforms per gene among blood samples remained low for both GRCh38 and T2T-CHM13 references (Figure 3B).

When the main variant categories are further separated into “Coding” and “Non-coding” we observe further differences between the references and among individuals (Figure 3C,D): the differences between GRCh38 and T2T-CHM13 in the number of variants identified for all categories in the “Coding” context (Figure 3C) show no differences from the overall counts (“Coding” plus “Non-coding”; Figure 1E,F) However, when the “Non-coding” subcategory was analyzed separately for each variant category (Figure 3D), GRCh38 generally identified a higher number of isoforms. Specifically, we see a higher difference in “FSM Non-coding”, “ISM Non-coding”, “NIC Non-coding”, and “Genic Intron Non-coding” when compared to the “Coding” subcategories (Figure 3C,D). When we analyzed the identified isoform number variability among individuals in the context of differences between the “Coding” and “Non-coding” subcategories, we observed an increase in the coefficient of variation in the “Non-coding” subcategories of “FSM”, “NNC”, “Antisense”, and “Fusion” when compared to the “Coding” subcategories (Figure 3C–E).

### 3.4. Features of Isoform Subcategories in Whole Blood

**Figure 4:**
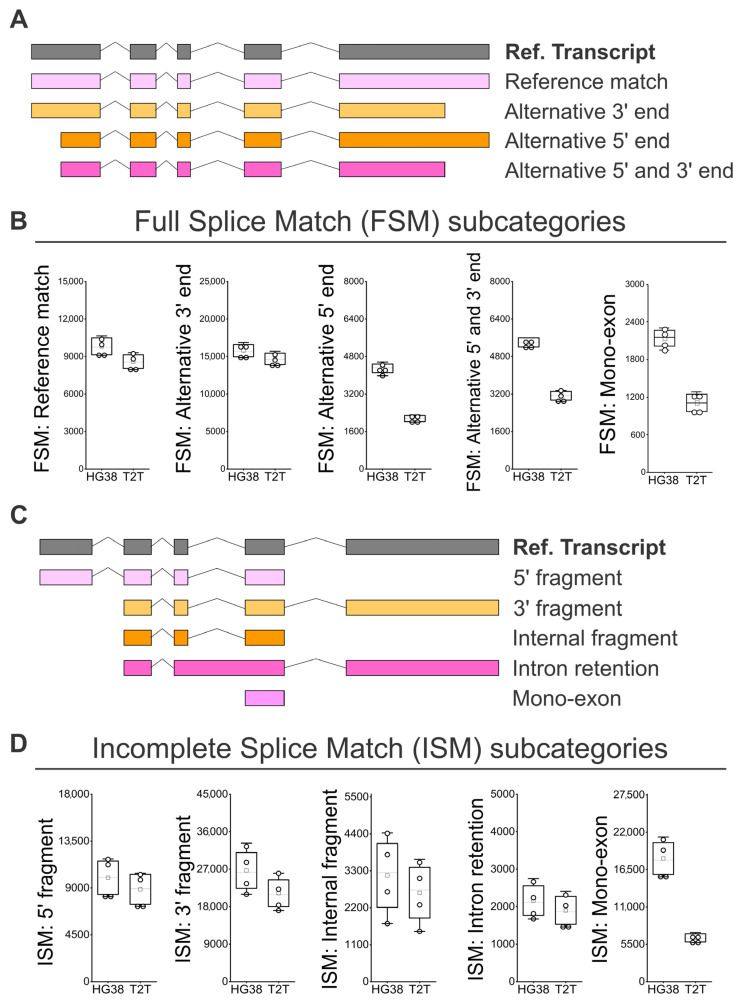
Identification of FSM and ISM isoform subcategories in peripheral blood using GRCh38 (HG38) and T2T-CHM13 (T2T) genome references. (**A**) SQANTI classification for subcategories of FSM isoforms [25,26]: “Alternative 5′ end”, “Alternative 5′ and 3′ end, and “Mono-exon”. (**B**) Number of isoforms by subcategories of FSM. (**C**) SQANTI classification for subcategories of ISM isoforms [25,26]: “5′ fragment”, “3′ fragment”, “Internal fragment”, “Intron retention”, and “Mono-exon”. (**D**) Number of isoforms by subcategories of ISM. (**B**,**D**) *N* = 4; white boxes show the interquartile range (25–75% percentile range), the median is designated by the horizontal line, and the mean is indicated by the white square. Boundaries of the whiskers are based on the 1.5 interquartile range value, and all observed data points outside the limit of the whiskers are considered outliers.

**Figure 5:**
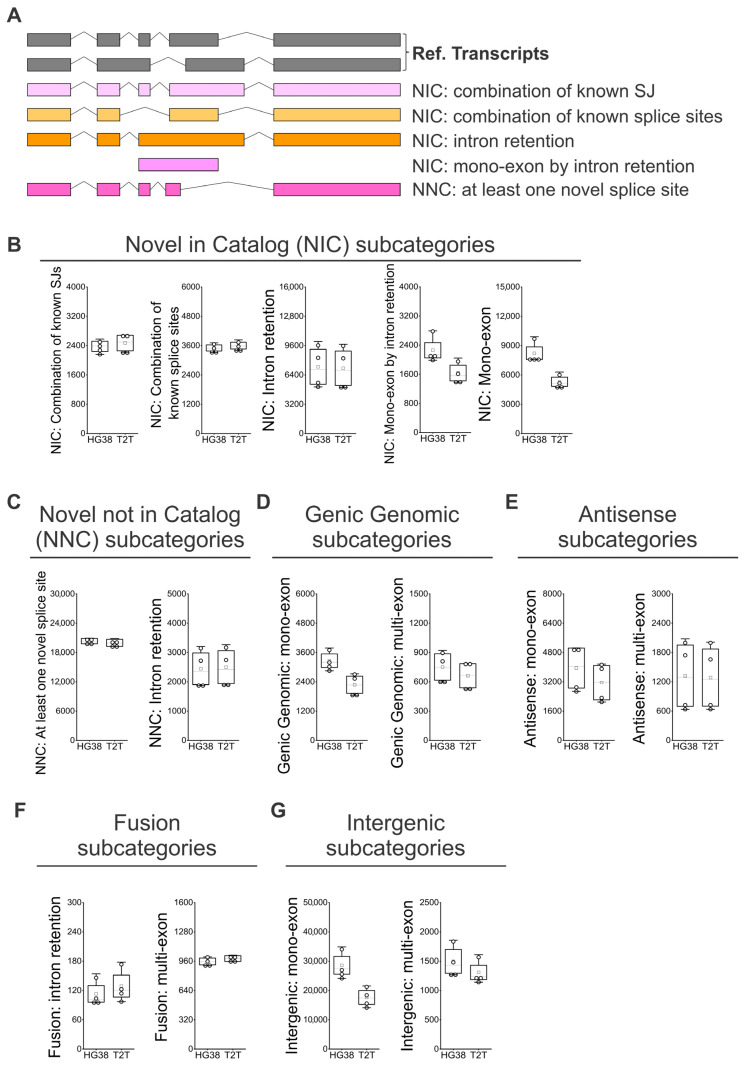
Identification of NIC and NNC isoform subcategories in peripheral blood using GRCh38 (HG38) and T2T-CHM13 (T2T) genome references. (**A**) SQANTI classification for subcategories of NIC and NNC isoforms [25,26]: “Combination of known SJs”, “Combination of known splice sites”, “Intron retention”, “Mono-exon by intron retention”, and “At least one novel splice site”. (**B**) Number of isoforms by subcategories of NIC. (**C**) Number of isoforms by subcategories of NNC. (**D**) Number of isoforms by subcategories of Genic Genomic. (**E**) Number of isoforms by subcategories of Antisense. (**F**) Number of isoforms by subcategories of Fusion. (**G**) Number of isoforms by subcategories of Intergenic. (**B**–**G**) *N* = 4; white boxes show the interquartile range (25–75% percentile range), the median is designated by the horizontal line, and the mean is indicated by the white square. Boundaries of the whiskers are based on the 1.5 interquartile range value, and all observed data points outside the limit of the whiskers are considered outliers.

Following the subcategorization analysis of FSM isoforms (Figure 4A) [25,26], we observed that the “Alternative 5′ end”, the “Alternative 5′ and 3′ end, and the “Mono-exon” are identified 1.7 to 2 times more often when we used GRCh38 than when we used T2T-CHM13; this points to limitations in the identification of alternative transcription start site (TSS) sequences of alternative 5′ ends when the T2T-CHM13 reference is used (Figure 4B). Regarding the ISM subcategories (Figure 4C), we observed a tendency to identify a higher number of isoforms when the GRCh38 reference was used. More strikingly, the number of recorded “Mono-exons” in the ISM subcategory was on average 2.8 higher when GRCh38 was compared to T2T-CHM13 (Figure 4D). Then, we analyzed the subcategories applied to novel isoforms (Figure 5A). No difference was observed between GRCh38 and T2T-CHM13 regarding the NIC subcategories “Combination of known SJs”, “Combination of known splice sites”, and “Intron retention”; while for the NIC subcategories “Mono-exon by intron retention” and “Mono-exon”, GRCh38 identified 1.4 and 1.6 times more isoforms than the T2T-CHM13 reference (Figure 5B). No difference was found in the number of identified NNC isoforms with “At least one novel splice site” or “Intron retention” (Figure 5C). Finally, regarding the categories “Genic Genomic”, “Antisense”, “Fusion”, and “Intergenic”, the main differences were generally related to the higher number of identified “mono-exons” by using GRCH38 (Figure 5D,E,G), while the “multi-exon” and “Fusion: intron retention” were characterized by similar numbers for both GRCh38 and T2T-CHM13 references (Figure 5D–G). In addition, we identified genes showing the highest diversity of full-length transcript isoforms in each sample and compared the results between the GRCh38 and T2T-CHM13 reference genomes. We found that the genes with the greatest isoform diversity were largely consistent across healthy individuals and between the two references (Supplementary Table S2). Notably, genes such as *AKAP13*, *PTPRC*, *LRRK2*, and *IQGAP1* are associated with the MAPK cascade, *FYB1*, *FCGR3B*, *ADGRE5*, *LILRB2*, *CTSS*, and *HLA-E* are involved in immune responses, and *PTPRC*, *LRRK2*, and *HLA-E* participate in the positive regulation of tumor necrosis factor production (Supplementary Table S3).

### 3.5. Expression Levels of lrRNA-Seq Identified Isoforms

**Figure 6:**
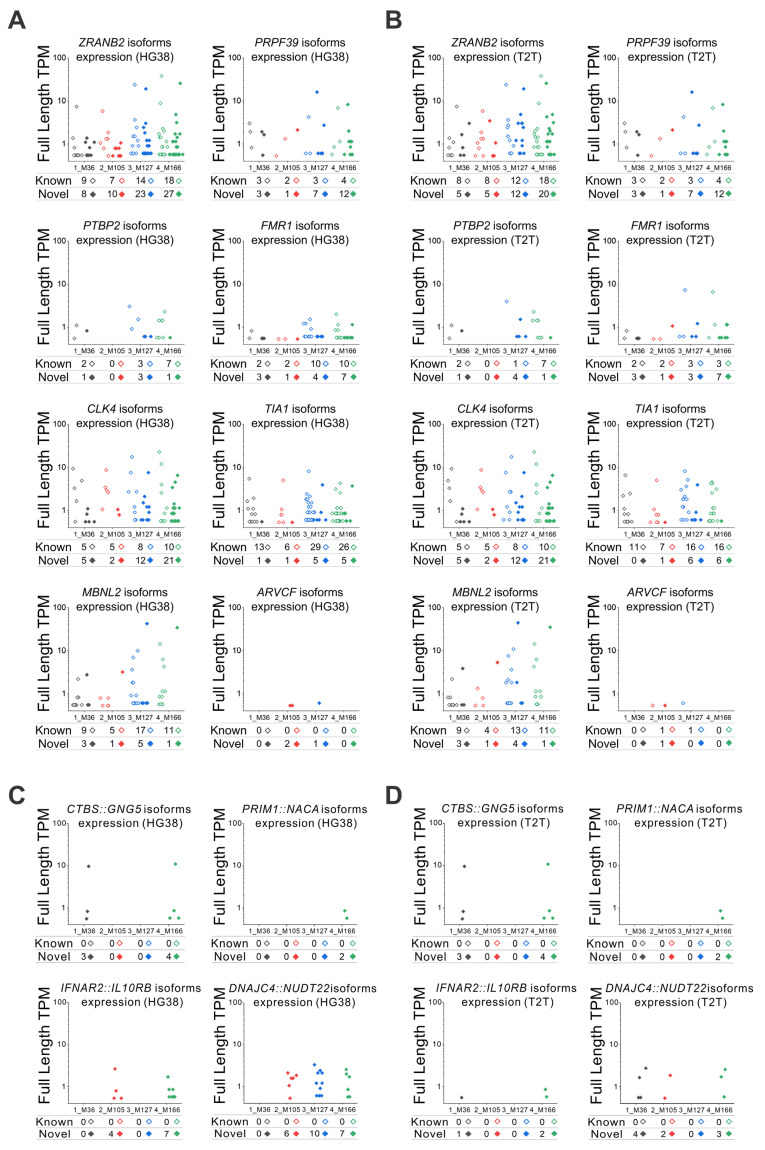
Long-read RNA-seq identified isoforms and expression levels. (**A**,**B**) Expression levels obtained by lrRNA-seq for each known and novel isoform of the selected eight genes, using both (**A**) GRCh38 (HG38) and (**B**) T2T-CHM13 (T2T) methods. Isoform expression of four fusion genes by (**C**) HG38 and (**D**) T2T methods: *CTBS*::*GNG5*, *PRIM1*::*NACA*, *IFNAR2*::*IL10RB*, and *DNAJC4*::*NUDT22*. The total number of isoforms and the TPM expression of each single isoform are shown in the graphs. The total numbers of known and novel isoforms are annotated for each blood sample at the base of the graphs.

We selected eight genes of interest that are related to mRNA splicing and modulation to verify the expression of each known and novel isoform when GRCh38 (Figure 6A) or T2T-CHM13 is used as the reference genome (Figure 6B). The expression in transcripts per million (TPM) of each single isoform for each gene and for each sample was obtained by lrRNA-seq, and the summation of all isoforms’ expressions corresponded to the overall expression of the considered gene. We can observe that the counts and expressions of known and novel isoforms can be very variable among genes. Moreover, within the analysis of the same gene, counts and expressions of isoforms are very different among individuals, even those with no apparent disease. The trend of overall expressions is similar whether the GRCh38 or T2T-CHM13 reference is used (Figure 6A,B). In some cases, for example in the analysis of the *FMR1* gene, in two samples (3_M127 and 4_M166), the number of isoforms identified by using GRCh38 (Figure 6A) is higher than when using T2T-CHM13 reference (Figure 6B), but on the other hand, the expression of one of the isoforms for the same gene for both 3_M127 and 4_M166 samples is about 10 times higher when T2T-CHM13 is used. In addition, since fusion genes are critical for the development of new diagnoses and new drugs, we selected four fusion genes for testing the isoform expression (Figure 6C,D): *CTBS*::*GNG5*, involved in nonneoplastic hematologic disorders in the lymph node and spleen, as well as in epithelial lesions in bladder, skin, and lung [30]; *PRIM1*::*NACA*, related to chronic myeloid leukemia [31]; *IFNAR2*::*IL10RB*, involved in lung nonneoplastic epithelial disorder [32]; and *DNAJC4*::*NUDT22*, related to lung adenocarcinoma [33]. Since the blood samples were from healthy individuals, we did not expect high levels of isoform expression related to these fusion genes. Nonetheless, we could confirm that the trend of detected expressions also approximately matched whether GRCh38 or T2T-CHM13 was used (Figure 6C,D). For *CTBS*::*GNG5* and *PRIM1*::*NACA*, the results were very similar, while for *IFNAR2*::*IL10RB* and *DNAJC4*::*NUDT22*, a certain degree of difference was noticed. We could also confirm the detection in blood of other isoforms derived from fusion genes specific to different organs, such as: *FOXP1*::*EIF4E3*, *ABI2*::*CYP20A1*, and *CTSD*::*IFITM10*, related to breast adenocarcinoma [33,34,35,36]; *HAVCR1*::*TIMD4* and *JAK3*::*INSL3*, related to kidney adenocarcinoma [34,36,37]; and *HACL1*::*COLQ* and *SIDT2*::*TAGLN*, related to lymph node nonneoplastic disorders [30,36].

These preliminary analyses need to be validated, since some of the fusion genes detected in healthy individuals might be artifacts. The expression identified by lrRNA-seq methods should be checked in terms of expression variations with other methods, such as DNA microarray or short-read RNA-seq. In this context, for the gene expression validation of the eight genes depicted in Figure 6A,B, we performed short-read RNA sequencing for all four samples, annotated with both GRCh38 and T2T-CHM13 references, and calculated the TPM values (Supplementary Figure S2).

The correlation between the genes’ expressions identified by short-read RNA-seq and the extent of expression identified for each gene/sample by lrRNA-seq (TPM expression of each single isoform together with the number of isoforms in their totality) was confirmed. These results confirm that the summation of isoforms’ expressions (overall gene expression) identified by lrRNA-seq methods can be cross-validated in terms of expression variations with short-read RNA-seq methods. In addition, for the eight novel isoforms shown in Figure 6A,B, we always used the sequence with the highest TPM value detected in each sample as the reference for mapping short-read RNA-seq data against both GRCh38 and T2T-CHM13 references. In cases where the sequence information was incomplete, we used regions shared across samples as the reference. The results of this mapping are summarized in Supplementary Figure S3. It should be noted that the novel isoforms identified by long-read sequencing largely overlap with the genomic regions of known genes. Therefore, most of these isoforms can also be detected by short-read sequencing, although detection may be limited for certain low-abundance isoforms, such as *MBNL2* (Supplementary Figure S3). To validate the fusion genes depicted in Figure 6C,D, we used the longest isoform detected for each of the four fusion genes, annotated against both GRCh38 and T2T-CHM13, as the reference sequence for short-read RNA-seq analysis. We confirmed that the long fusion isoforms identified by long-read sequencing were also detectable with short reads, as the reads mapped to multiple regions within the fusion (Supplementary Figure S4).

## 4. Discussion

Blood flows all through the body and is in constant contact with every organ via the cardiovascular system. It consists of many different cell types, which differ in the amount of RNA they contain; this influences the phenotype and function of these cells [38], and these changes reflect the physiological or pathological condition of the host [39,40]. A large percentage of potential novel RNA isoforms are still undiscovered and need to be characterized [3]. Isoform variations and differences in splicing are key factors to be investigated. The lrRNA-seq analysis allows us to identify new isoforms as well as their expression, which could be associated with disease risk [41,42], clarifying how the transcriptome performs in response to pathological stimuli [43]. In this context, the identification of new RNA isoforms and their relative expression analysis in whole blood is an important means to evaluate an individual’s biological condition and assess unmanifested diseases. Therefore, we used full blood from four healthy individuals with no apparent disease and examined their RNA variant composition and expression using long-read RNA-seq. Two different human reference genome mapping techniques, GRCh38 and T2T-CHM13, were used to assess the lrRNA-seq analysis performance and downstream results. We found that the Iso-Seq data analyzed with GRCh38 could identify about 46,000 genes, 1.3-fold more genes than T2T-CHM13. The difference was mainly due to the higher number of identified novel genes; a similar increase was confirmed for identified unique isoforms. Among all the isoforms identified, 47% were the novel isoforms when GRCh38 was used, and 50% were the novel isoforms when T2T-CHM13 was used. Independent of the reference gene used, a large number of novel isoforms can still be discovered and characterized in full blood. Regarding novel isoforms, similar numbers were identified for “NIC”, “NNC”, “Antisense”, and “Fusion”, while the largest difference in isoform numbers between GRCh38 and T2T-CHM13 were for the subcategories “Genic Genomic”, “Intergenic”, “Genic Intron”, and “Mono-Exon”. For all of these, GRCh38 allowed the identification of a higher number when compared to T2T-CHM13. When T2T-CHM13 was used as the reference, we noticed a reduced ability to identify isoforms aligning in the intron and intergenic regions. This was unexpected, since we thought T2T-CHM13 would perform better than GRCh38 due to being a more gapless sequence reference of the human genome [8]. Interestingly, we observed no discrepancies in the recognition of splice junctions, where both GRCh38 and T2T-CHM13 performed similarly, so we deduced that the above differences were a matter of sequence alignment in the non-exonic areas. Another difference between GRCh38 and T2T-CHM13 was observed when following the subcategorization analysis of FSM and ISM isoforms. In particular, T2T-CHM13 was outperformed by GRCh38 in the identification of isoforms with alternative 5′ ends or with a missing exon at the 5′ end. The subcategories “Antisense”, “NIC”, “Genic Genomic”, and “Intergenic” had the highest variability in the number of isoforms detected among individuals; it follows that the isoforms within these subcategories were the most related to physiological differences among individuals. In this context, “Antisense” isoforms could be used as drug targets since they regulate gene expression and are involved in cancer promotion or suppression [44,45], while novel “Genic Genomic”, and “Intergenic” isoforms may also regulate gene expression as well as chromatin remodeling [46,47]. In addition, similar statistics for both GRCh38 and T2T-CHM13 were obtained, indicating that the interindividual differences were due to the blood features and not to the selected reference genome. Lastly, we studied the expression of eight selected genes of interest related to mRNA splicing, maturation, and process stability as well as four fusion genes related to diseases. Among genes, the counts and expressions of known and novel isoforms were very variable, but unexpectedly, also within the same gene, counts and expressions of isoforms were very different among individuals even in the absence of apparent disease. In terms of gene expression, similar levels could be observed independent of the reference genome used. For some genes, the known/novel ratio of the number of isoforms is very high; one such example is TIA1, which is an extensively studied gene (1210 hits on PubMed [27 August 2024]) [48]. Meanwhile, for the gene ZRANB2, a much less investigated gene (39 hits on PubMed [27 August 2024]) [48], many novel isoforms were identified, in contrast to the known isoforms. We confirmed that blood examination would allow the identification of isoforms derived from fusion genes for both hematological diseases and organ-specific diseases. For long-read RNA-seq, it is also necessary to assess and confirm that the RNA information is correct by using other techniques, and to identify which reference assembly performs better and in which conditions. GRCh38 generally identifies a higher number of isoforms when compared to T2T-CHM13, but regions of the GRCh38 reference are sometimes artificial, with incomplete assembly or incorrect sequences [49,50,51], and this may have led to the identification of a higher number of false positives. In this regard, we confirmed that the validation of long-read RNA-seq information can be performed by short-read RNA-seq with both GRCh38 and T2T-CHM13 references for both the detection and the quantification of genes and the detection of fusion genes. The correlation between the genes’ expressions identified by short-read RNA-seq and the expressions identified for each gene/sample by lrRNA-seq was confirmed. When the sequence information was incomplete, we used regions shared across samples as the reference. Short-read RNA-seq may fail to fully detect the isoform due to insufficient read length or fragmentation; nonetheless, even low-abundance isoforms are still detectable, and the detection sensitivity may differ between GRCh38 and T2T-CHM13 references. Issues related to isoform identification can occur due to blood cell heterogeneity—cell type composition varies between individuals, especially when patients are in disease states, so the proportions of immune cell types might affect the transcriptome and potentially induce mistakes in biomarker identification. This complexity means that analysis in the future needs to take account of cellular heterogeneity for proper interpretation. Computational deconvolution methods, such as BayesPrism, can estimate the proportions of different cell types and help to interpret bulk RNA-seq data more accurately [1,52]. Gender differences might also impact the analysis of long-read RNA sequencing. However, with only four samples in this pilot study (two males and two females), it is premature to perform any stratified analysis. Previous transcriptome studies with short-read RNA-seq of whole blood [53] and in immune cells [54] have reported variation in expression of immune response genes, cell cycle regulation, and oxidative stress. Future studies with larger sample sizes will be needed to assess potential gender effects reliably. Another limitation is the matter of selection bias—since blood samples are derived from participants of Japanese ethnicity, the data obtained should be carefully examined before conclusions are generalized for an international context. From a statistical perspective, four samples represent a small cohort, and these findings should be considered an initial pilot study. Broader validation across larger and more diverse populations is needed to definitively determine which reference genome, GRCh38 or T2T-CHM13, is preferable.

## 5. Conclusions

In this preliminary assessment of the whole blood full transcriptome, GRCh38 annotation appears to identify a higher number of isoforms when compared to T2T-CHM13. These differences are more evident for regions related to intron and intergenic sequencies, as well as for the identification of mono-exons. Moreover, knowing how these gene products are generated is crucial, for example in cases of isoforms derived from fusion genes, which are important when investigating diseases. We performed this first analysis to create a database that will allow users to view different isoforms, associated exons, and the endpoints of transcription on a map with two different genome references.

## Data Availability

Original dataset available upon reasonable request from the authors.

## Supplementary Materials

The following supporting information can be downloaded at: https://www.mdpi.com/article/10.3390/genes16091075/s1, Table S1: Clinical parameters of the 4 individuals whose blood was examined for gene expression and isoform identification; Table S2: Identified genes with the highest number of diverse full-length transcript isoforms for each sample: comparison between the two reference genomes GRCh38 and T2T-CHM13; Table S3: Enrichment Analysis by Gene Ontology Biological Process of identified genes with the highest number of diverse full-length transcript isoforms in blood of healthy individuals (Supplementary Table S2); Figure S1: Transcriptomic profiles depicted by PCA; Figure S2: Gene expression validation by short-read RNA sequencing of the 8 genes depicted in Figure 6A,B; Figure S3: Short-read RNA-seq isoform validation of the 8 genes depicted in Figure 6A,B; Figure S4: Short-read RNA-seq validation of the 4 fusion genes depicted in Figure 6C,D.

## Abbreviations

The following abbreviations are used in this manuscript:

CV	Coefficient of Variation
FSM	Full Splice Match
GRCh38	Genome Reference Consortium Human Build 38
GTF	Gene Transfer Format
ISM	Incomplete Splice Match
lrRNA-seq	long-read RNA sequencing
NIC	Novel in Catalog
NNC	Novel Not in Catalog
SJs	Splice Junctions
T2T	Telomere-to-Telomere
T2T-CHM13	Telomere-to-Telomere assembly of the CHM13 cell line
TPM	Transcripts per Million
TSS	Transcription Start Site
