# Supplementary material for: Evaluation of Long-Read RNA Sequencing Procedures for Novel Isoform Identification and Quantification in Human Whole Blood
Source: Genes (Basel). 2025 Sep 12;16(9):1075. doi: 10.3390/genes16091075 (PMC12469794; doi:10.3390/genes16091075)
Supplement: Supplementary file 1 [file genes-16-01075-s001.zip › genes-3837191-supplementary.pdf]

Evaluation of long-read RNA sequencing procedures for novel isoforms identification and quantification in human whole blood

SUPPLEMENTARY INFORMATION

1. Supplementary Materials and Methods

1.1. Whole blood RNA isolation and short-read RNA-seq analysis

Total RNA was isolated from whole blood of the same patients (n = 4) as described in the paragraph “2.2. Whole blood RNA isolation and lrRNA-seq library creation” within the main manuscript. The total blood RNA was prepared by DNA Chip Lab (Tokyo, Japan) for short-read RNA-seq using NEBNext® Ultra II RNA Directional Library prep for Illumina kit (Illumina, Inc. San Diego, CA, USA). The library-conditioned samples were read for RNA sequences by NextSeq (Illumina, Inc.) at Kazusa DNA Research Institute (Chiba, Japan). The resulting FASTQ files were used to validate the eight isoforms and four fusion genes shown in Figure 6. For isoform validation, the most highly expressed transcript within each sample was selected as the reference, and raw sequence reads were mapped using CLC Genomics Workbench (Qiagen, v23.0). Reads from both short- and long-read platforms were aligned to T2T-CHM13 v2.0 reference genome as well as to the latest GRCh38 (hg38, primary assembly, GCA_000001405.30) reference genome. For fusion gene validation, the transcript detected in each sample was considered identical across databases, with differences only in transcript length; the longest sequence for each fusion gene was selected as the reference and mapped separately to T2T and GRCh38.

2. Supplementary Tables

Supplementary Table S1. Clinical parameters of the 4 individuals whose blood was examined for gene expression and isoform identification.

Clinical parameters	1_M_36	2_M_105	3_M_127	4_M_166
Sex	Male	Female	Female	Male
Age	57	57	73	48
Height (cm)	182.5	166.1	152.2	170.8
Weight (kg)	73.1	55.5	52.5	79.2
BMI (kg/m^2)	22	20.2	22.7	27.2
Systolic blood pressure (mmHg)	118	116	130	107
Diastolic blood pressure (mmHg)	75	78	81	64
HbA1c (NGSP; %)	6.2	5.6	5.6	5.5
Total cholesterol (mg/dl)	233	248	265	185
Triglycerides (mg/dl)	211	62	63	114
High Density Lipoprotein (mg/dl)	37	85	89	63
Low Denstiy Lipoprotein (mg/dl)	164	143	149	101
AST (IU/L)	22	21	20	15
ALT (IU/L)	35	15	10	16
γGTP (U/L)	36	22	10	74
Creatinine (mg/dl)	0.91	0.71	1.21	1.00
White Blood Cell count (10^2/μL)	38.8	54.1	46.6	48.5
Red Blood Cell count (10^4/μL)	532	459	400	473
Hemoglobin (g/dl)	15.5	14.3	11.4	13.8
Hematocrit (%)	46.8	44	36.9	42.3
Platelet count (10^4/μL)	19.8	15.8	31.2	30.5

BMI, body mass index; HbA1c, Hemoglobin A1c; AST, aspartate aminotransferase; ALT, alanine aminotransferase; γGTP, γ-glutamyl transferase.

Supplementary Table S2. Identified genes with the highest number of diverse full-length transcript isoforms for each sample: comparison between the two reference genomes GRCh38 and T2T-CHM13.

GRCh38								T2T-CHM13							
1_M_36		2_M_105		3_M_127		4_M_166		1_M_36		2_M_105		3_M_127		4_M_166	
Gene	N. of isoforms	*Gene*	N. of isoforms	*Gene*	N. of isoforms	*Gene*	N. of isoforms	*Gene*	N. of isoforms	*Gene*	N. of isoforms	*Gene*	N. of isoforms	*Gene*	N. of isoforms
RIPOR2	364	*RIPOR2*	354	*RIPOR2*	390	*RIPOR2*	291	*CSF3R*	276	*CSF3R*	250	*RIPOR2*	251	*PTPRC*	218
CSF3R	335	*CSF3R*	318	*PTPRC*	330	*PTPRC*	281	*PTPRC*	221	*RIPOR2*	215	*PTPRC*	241	*RIPOR2*	193
PTPRC	282	*PTPRC*	262	*FYB1*	262	*NAMPT*	248	*RIPOR2*	216	*PTPRC*	214	*CSF3R*	192	*CSF3R*	172
SORL1	225	*FYB1*	226	*SORL1*	246	*CSF3R*	207	*CELF2*	142	*HLA-E*	168	*FYB1*	176	*NAMPT*	143
SLC25A37	224	*SORL1*	210	*NAMPT*	236	*FYB1*	197	*FYB1*	137	*FYB1*	151	*CELF2*	153	*FYB1*	142
FYB1	214	*AKAP13*	209	*CELF2*	234	*SLC25A37*	193	*CTSS*	131	*ADGRE5*	140	*SMCHD1*	146	*PCBP2*	128
CELF2	209	*HLA-E*	199	*CSF3R*	231	*CELF2*	173	*HLA-E*	128	*MYO1F*	140	*HLA-E*	131	*RNF10*	127
FCGR3B	200	*SLC25A37*	199	*SLC25A37*	191	*SMCHD1*	171	*HNRNPK*	128	*PCBP2*	138	*LRRK2*	131	*SMCHD1*	121
NIBAN1	175	*CELF2*	196	*SMCHD1*	186	*MALAT1*	157	*MYO1F*	128	*CELF2*	134	*MYO1F*	131	*CELF2*	120
RNF10	174	*NIBAN1*	192	*LRRK2*	172	*RNF10*	153	*CTSB*	127	*SYNE2*	127	*LILRB2*	128	*HNRNPK*	119
AKAP13	169	*RNF213*	173	*RNF149*	171	*DMTN*	149	*PCBP2*	125	*RNF213*	122	*SORL1*	126	*USP15*	118
VCAN	164	*AOAH*	172	*LRRFIP1*	163	*SORL1*	149	*DMTN*	123	*TCIRG1*	120	*PCBP2*	125	*ARHGAP15*	116
WIPF1	163	*ADGRE5*	164	*WIPF1*	162	*FCGR3B*	148	*SMCHD1*	117	*SORL1*	114	*HNRNPK*	124	*HLA-E*	115
NAMPT	157	*MYO1F*	163	*HLA-E*	155	*RNF149*	145	*ADGRE5*	114	*AOAH*	110	*RNF10*	123	*DMTN*	112
SMCHD1	157	*PCBP2*	155	*NIBAN1*	154	*JAML*	143	*IQGAP1*	108	*AKAP13*	107	*WIPF1*	121	*WIPF1*	112
CTSS	155	*TXNIP*	152	*JAML*	152	*LRRFIP1*	142	*SORL1*	108	*CAST*	106	*TCIRG1*	119	*JAML*	110
DMTN	154	*CFLAR*	145	*RNF10*	152	*WIPF1*	142	*BAG6*	107	*HLA-A*	105	*TRAF3IP3*	118	*VMP1*	109
JAML	154	*IQGAP1*	143	*IKZF1*	147	*PCBP2*	141	*LILRB2*	106	*CTSB*	104	*NPRL3*	110	*TRAF3IP3*	107
HLA-E	150	*IKZF1*	142	*DOCK8*	145	*VMP1*	139	*LILRA1*	105	*IQGAP1*	104	*DMTN*	108	*NPRL3*	106
IQGAP1	150	*CAST*	139	*MBNL1*	145	*LRRK2*	137	*LOC124905743*	102	*LILRB2*	104	*JAML*	108	*CTSB*	103

Supplementary Table S3. Enrichment Analysis by Gene Ontology Biological Process of identified genes with the highest number of diverse full-length transcript isoforms in blood of healthy individuals (Supplementary Table S2).

GOTERM_BP Term	Count	*P*-Value	*Genes*
GO:0000165~MAPK cascade	4	0.0014	*AKAP13, PTPRC, LRRK2, IQGAP1*
GO:0006955~immune response	6	0.0026	*FYB1, FCGR3B, ADGRE5, LILRB2, CTSS, HLA-E*
GO:0045860~positive regulation of protein kinase activity	3	0.0026	*PTPRC, LRRK2, IQGAP1*
GO:0042130~negative regulation of T cell proliferation	3	0.0036	*RIPOR2, LILRB2, HLA-E*
GO:0032760~positive regulation of tumor necrosis factor production	3	0.0172	*PTPRC, LRRK2, HLA-E*
GO:0097067~cellular response to thyroid hormone stimulus	2	0.0178	*CTSS, CTSB*
GO:0007166~cell surface receptor signaling pathway	4	0.0241	*FCGR3B, PTPRC, ADGRE5, LILRB2*
GO:0007165~signal transduction	7	0.0275	*CSF3R, HNRNPK, PTPRC, NAMPT, LILRB2, IQGAP1, HLA-E*
GO:0002639~positive regulation of immunoglobulin production	2	0.0526	*PTPRC, HLA-E*

3. Supplementary Figures

Supplementary Figure S1

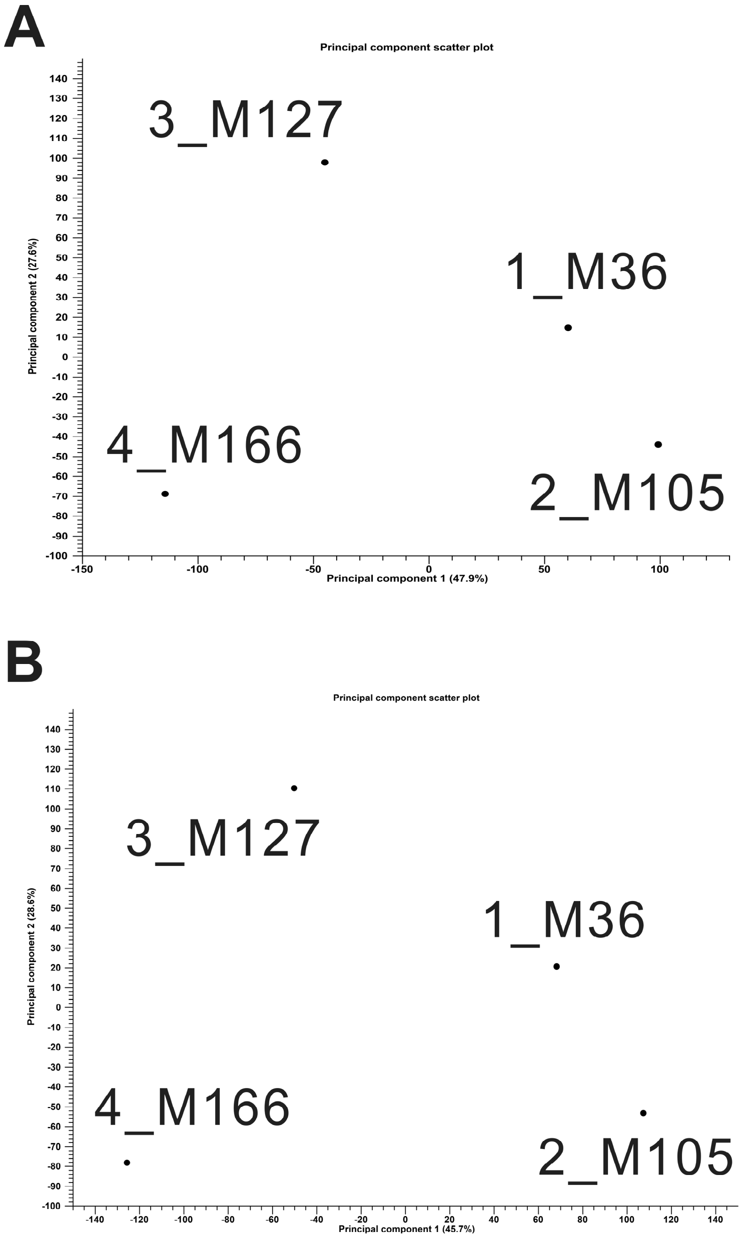

Supplementary Figure S1. Transcriptomic profiles depicted by PCA. (A) PCA of GRCh38-annotated data for four samples, showing biological variability. (B) PCA of T2T-CHM13-annotated data for four samples, showing biological variability.

Supplementary Figure S2

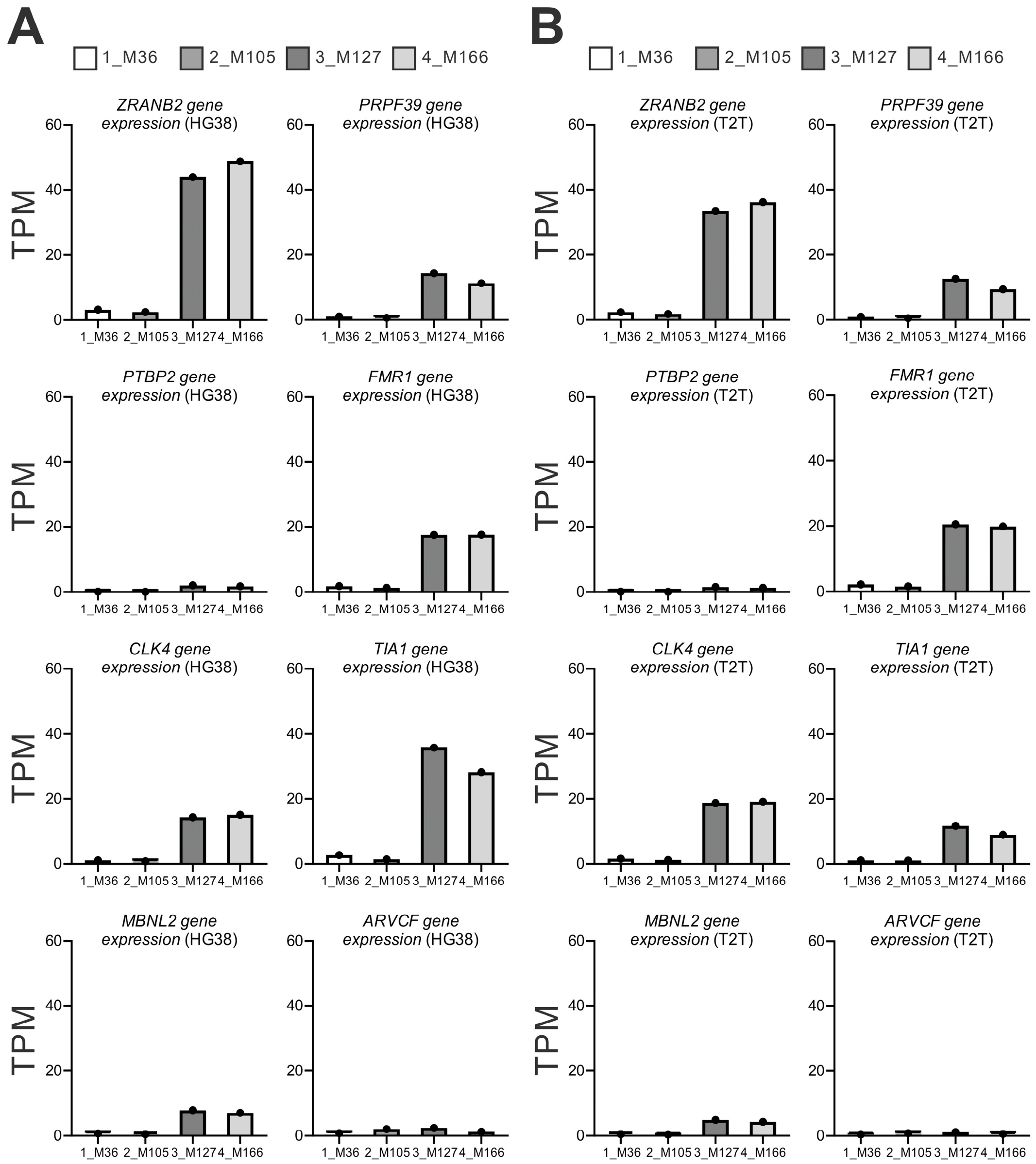

Supplementary Figure S2. Gene expression validation by short-read RNA sequencing of the 8 genes depicted in Figure 6A, B. Data from short-read RNA sequencing for all 4 blood samples, annotated with both (A) GRCh38 and (B) T2T-CHM13 references. Y-axis: TPM (Transcripts Per Million); TPM is calculated in a way that normalizes for both gene length and sequencing depth, the distribution of read lengths within a sample does not directly affect TPM.

Supplementary Figure S3

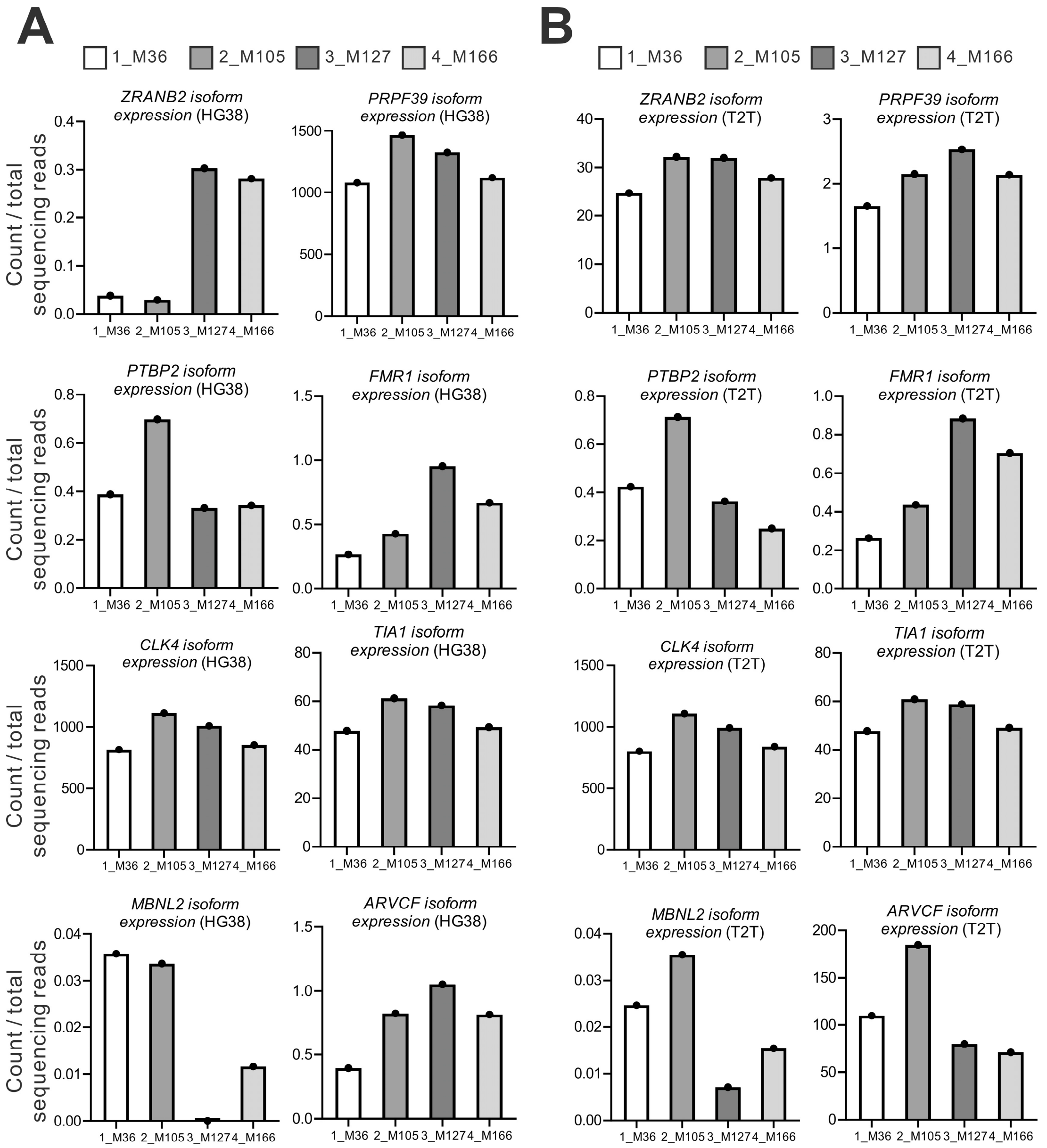

Supplementary Figure S3. Short-read RNA-seq isoform validation of the 8 genes depicted in Figure 6A, B. The sequence with the highest TPM value detected in each sample by long-read RNA-seq was used as the reference for mapping short-read RNA-seq data against both (A) GRCh38 and (B) T2T-CHM13 references. In cases where sequence information was incomplete, we

used regions shared across samples as the reference. The counts were normalized by comparing with the total number of sequencing reads per sample (count / total sequencing reads).

Supplementary Figure S4

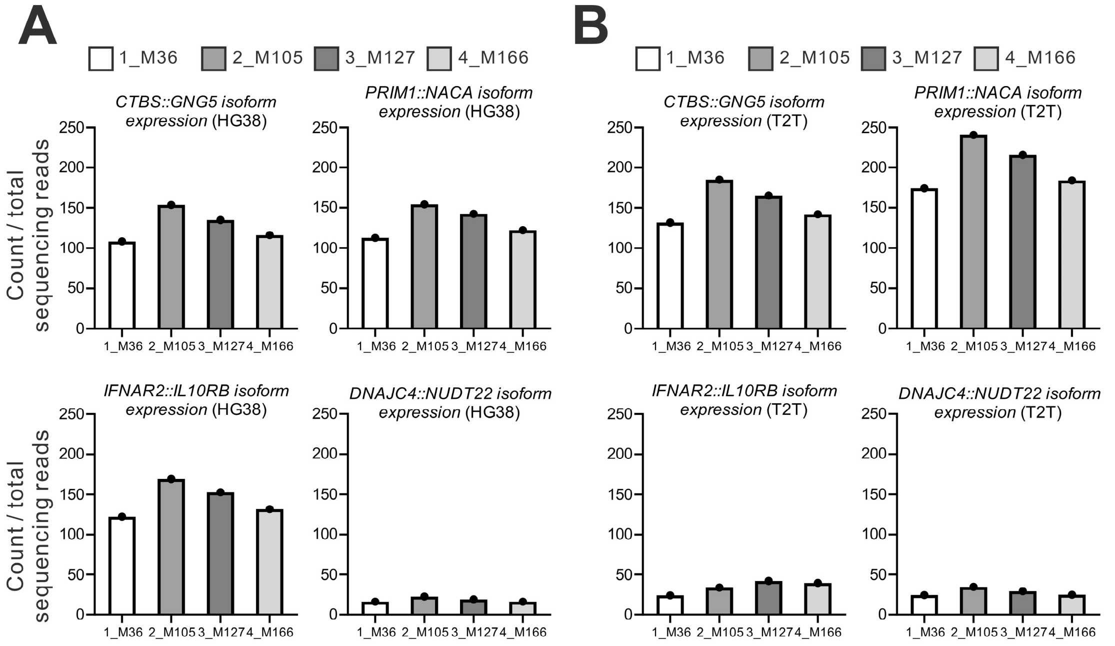

Supplementary Figure S4. Short-read RNA-seq validation of the 4 fusion genes depicted in Figure 6C, D. The longest isoform detected by long-read RNA-seq for each of the four fusion genes was chosen and annotated against both (A) GRCh38 and (B) T2T-CHM13, this was used as reference sequence for short-read RNA-seq analysis. For the detection of fusion genes, the mapping counts depend on the read length and library size. The counts were normalized by comparing with the total number of sequencing reads per sample (count / total sequencing reads). This allows for relative comparisons between samples despite differences in total read numbers.
